# Direct Stenting versus Conventional Stenting in Patients with ST-Segment Elevation Myocardial Infarction—A COMPARE CRUSH Sub-Study

**DOI:** 10.3390/jcm12206645

**Published:** 2023-10-20

**Authors:** Rosanne F. Vogel, Ronak Delewi, Jeroen M. Wilschut, Miguel E. Lemmert, Roberto Diletti, Ria van Vliet, Nancy W. P. L. van der Waarden, Rutger-Jan Nuis, Valeria Paradies, Dimitrios Alexopoulos, Felix Zijlstra, Gilles Montalescot, Dominick J. Angiolillo, Mitchell W. Krucoff, Nicolas M. Van Mieghem, Pieter C. Smits, Georgios J. Vlachojannis

**Affiliations:** 1 Department of Cardiology, University Medical Center Utrecht, 3584 CX Utrecht, The Netherlands; 2 Department of Cardiology, Amsterdam UMC Location AMC, University of Amsterdam, 1105 AZ Amsterdam, The Netherlands; 3 Department of Cardiology, Erasmus University Medical Center, 3000 CA Rotterdam, The Netherlands; 4 Department of Cardiology, Isala Hospital, 8025 AB Zwolle, The Netherlands; 5 Department of Cardiology, Maasstad Hospital, 3079 DZ Rotterdam, The Netherlands; 6 AmbulanceZorg Rotterdam-Rijnmond, 2993 LT Barendrecht, The Netherlands; 7 Department of Cardiology, Attikon University Hospital, 12462 Athens, Greece; 8 ACTION Group, Groupe Hospitalier Pitie-Salpetriere Hospital (AP-HP), Sorbonne University, 75013 Paris, France; 9 Division of Cardiology, University of Florida College of Medicine, Jacksonville, FL 32610, USA; 10 Department of Cardiology, Duke University Medical Center, Durham, NC 27710, USA; 11 Department of Cardiology, Euroclinic Athens, 11521 Athens, Greece

**Keywords:** ST-segment elevation myocardial infarction, primary percutaneous coronary intervention, pretreatment, direct stenting

## Abstract

Background: Direct stenting (DS) compared with conventional stenting (CS) after balloon predilatation may reduce distal embolization during percutaneous coronary intervention (PCI), thereby improving tissue reperfusion. In contrast, DS may increase the risk of stent underexpansion and target lesion failure. Methods: In this sub-study of the randomized COMPARE CRUSH trial (NCT03296540), we reviewed the efficacy of DS versus CS in a cohort of contemporary, pretreated ST-segment elevation myocardial infarction (STEMI) patients undergoing primary PCI. We compared DS versus CS, assessing (1) stent diameter in the culprit lesion, (2) thrombolysis in myocardial infarction (TIMI) flow in the infarct-related artery post-PCI and complete ST-segment resolution (STR) one-hour post-PCI, and (3) target lesion failure at one year. For proportional variables, propensity score weighting was applied to account for potential treatment selection bias. Results: This prespecified sub-study included 446 patients, of whom 189 (42%) were treated with DS. Stent diameters were comparable between groups (3.2 ± 0.5 vs. 3.2 ± 0.5 mm, *p* = 0.17). Post-PCI TIMI 3 flow and complete STR post-PCI rates were similar between groups (DS 93% vs. CS 90%, adjusted OR 1.16 [95% CI, 0.56–2.39], *p* = 0.69, and DS 72% vs. CS 58%, adjusted OR 1.29 [95% CI 0.77–2.16], *p* = 0.34, respectively). Moreover, target lesion failure rates at one year were comparable (DS 2% vs. 1%, adjusted OR 2.93 [95% CI 0.52–16.49], *p* = 0.22). Conclusion: In this contemporary pretreated STEMI cohort, we found no difference in early myocardial reperfusion outcomes between DS and CS. Moreover, DS seemed comparable to CS in terms of stent diameter and one-year vessel patency.

## 1. Introduction

Primary percutaneous coronary intervention (PCI) is the preferred revascularization strategy in patients presenting with ST-segment elevation myocardial infarction (STEMI), and it is highly effective in restoring the epicardial patency of the infarct-related artery [1]. Notwithstanding, reperfusion at the tissue level in the infarct-related area is often deficient, leading to suboptimal myocardial salvage and poor clinical outcomes [2,3,4]. Incomplete tissue level reperfusion in patients treated with primary PCI is being attributed—in addition to injury through myocardial ischemia and reperfusion—to distal embolization of culprit lesion-related atherothrombotic material and subsequent downstream microvascular obstruction.

Direct stenting (DS) without preceding balloon dilatation is a mechanical reperfusion technique that avoids unnecessary manipulation of the culprit lesion and aims to reduce vessel damage, distal embolization, and microvascular obstruction in STEMI patients undergoing primary PCI [5,6,7]. The efficacy of this strategy has been investigated in several randomized trials over the last two decades reviewing early epicardial and myocardial reperfusion and clinical outcomes [7,8,9]. However, these trials reported conflicting results in terms of epicardial and myocardial reperfusion benefits. Moreover, these trials were conducted before the introduction of several pharmacological and mechanical reperfusion advances (e.g., potent oral P2Y_12_ inhibitors, second/third generation drug-eluting stents, and the differentiated use of glycoprotein inhibitors and thrombus-aspiration). Furthermore, the efficacy of DS versus CS in STEMI patients with a high thrombus burden at their initial angiography—assumed to represent a high risk of distal embolization and microvascular obstruction—has not been fully elucidated.

The randomized COMPARE CRUSH (Comparison of Pre-hospital Crushed Versus Uncrushed Prasugrel Tablets in Patients With STEMI Undergoing Primary Percutaneous Coronary Interventions) trial assessed the efficacy of pre-hospital administration of crushed compared with the integral prasugrel loading dose in consecutive STEMI patients who were managed by the emergency medical service before undergoing primary PCI [10,11]. In the present sub-study, we aim to investigate the efficacy of DS versus CS in STEMI patients enrolled in the COMPARE CRUSH trial.

## 2. Materials and Methods

### 2.1. Study Design and Population

The randomized COMPARE CRUSH trial (Clinical Trial Identifier NCT03296540) was an investigator-initiated, multicenter ambulance trial conducted in the Netherlands between 2017 and 2021. The trial was conducted in accordance with the Declaration of Helsinki (64th World Medical Association General Assembly, Fortaleza, Brazil, October 2013), the Medicinal Research Involving Human Subjects Act, and the International Conference on Harmonisation Good Clinical Practice. The study protocol and all study procedures were approved by the local ethics committee. All participants who were included in the analyses provided informed consent.

The trial design and study procedures have been previously described [10]. In brief, patients were eligible for study participation if myocardial infarction symptoms had started less than six hours before first medical contact and if new ST-segment deviations consistent with STEMI were present on the 12-lead ECG. Main exclusion criteria included contra-indications for prasugrel treatment (including hypersensitivity, previous stroke, and recent surgery), an indication for chronic anticoagulant therapy, and presentation with cardiogenic shock or cardiac arrest. Eligible patients were randomized at first medical contact by the emergency medical service to receive a 60 mg prasugrel loading dose administered as either crushed or integral tablets, in addition to standard pharmacological treatment consisting of 500 mg aspirin (intravenous) and 5.000 units of unfractionated heparin. Patients were transferred to an interventional center to undergo diagnostic angiography and primary PCI as indicated, according to standard practice. PCI-related strategies, including the use of manual thrombus aspiration devices and the use of glycoprotein IIb/IIIa inhibitors as a bailout therapy, were implemented at the discretion of the operator. The main trial’s primary study endpoints were two surrogate markers of early myocardial reperfusion: thrombolysis in myocardial infarction (TIMI) 3 flow in the infarct-related artery (IRA) pre-PCI, and complete (≥70%) ST-segment resolution (STR) measured one hour post-PCI. The main trial’s secondary outcomes included clinical outcomes at 30 days and at one year. Crushed compared with integral prasugrel loading dose administration in STEMI patients undergoing primary PCI failed to facilitate improved early myocardial reperfusion, despite a significantly stronger, faster onset of antiplatelet effect in patients treated with crushed prasugrel [11,12]. Moreover, there was no apparent clinical benefit of crushed compared with integral prasugrel at 30-day and one-year follow-ups [13].

For the present sub-study, we included a total of 594 patients enrolled in the COMPARE CRUSH trial who underwent primary PCI after initial angiography. Additional exclusion criteria were (i) patients treated by low-volume operators (i.e., conducting less than ten study inclusion procedures during the total enrollment period, *n* = 9), (ii) cases in which manual thrombus-aspiration was performed prior to PCI (*n* = 94), and/or cases in which glycoprotein IIb/IIIa inhibitors bailout was used (*n* = 45). The latter two patient groups were excluded because of the possible interaction of these additional interventions with the DS/CS approach. The final sub-study cohort comprised 446 patients (DS *n* = 189, CS *n* = 257).

### 2.2. Definitions and Endpoints

Patients were stratified into two groups based on the PCI strategy that was used (DS or CS). DS was defined as stent implantation in the infarct-related artery without previous balloon pre-dilatation. High thrombus burden was defined as TIMI thrombus grades 3–5 in the IRA on initial angiography and was graded by an independent blinded core laboratory.

We assessed early myocardial reperfusion endpoints and clinical outcomes between groups. Early myocardial reperfusion endpoints included angiographic parameters (TIMI flow in the IRA post-PCI, TIMI myocardial blush grade in the IRA post-PCI, and corrected TIMI frame count [cTFC] post-PCI) and the ECG parameter complete STR one hour post-PCI. Optimal cTFC was defined as ≤23 frames per second (FPS) [2]. Angiographic and ECG endpoints were analyzed by a blinded, independent core laboratory. Clinical outcomes included mortality, myocardial re-infarction, stent thrombosis, stroke, and urgent revascularization at one year, and they were adjudicated by a blinded, independent clinical events committee.

### 2.3. Statistical Analysis

IBM SPSS Statistics (version 26.0.0.1) software was used to conduct the statistical analyses, and GraphPad Prism (version 9) software was used to create figures. We compared early myocardial reperfusion endpoints and clinical outcomes between the DS and CS groups using univariate logistic regression. Results were reported as crude odds ratios (ORs) with 95% confidence intervals (CIs), and *p*-values of less than 0.05 were considered to be statistically significant. In the case of rare events with expected event counts of less than five in each category, we used a Fisher’s exact test to compute *p*-values.

To minimize the potential bias associated with the observational design of this sub-study, we additionally performed a propensity score weighted logistic regression analysis. Multiple imputation methods were applied to estimate missing data needed to calculate propensity scores and were performed under Rubin’s assumption that missing data was distributed randomly. The propensity scores for the choice of PCI strategy (DS or CS) were based on baseline and procedural characteristics including age, sex, ethnicity, weight, height, hypertension, diabetes, dyslipidemia, smoking, family history of cardiovascular disease, previous PCI, previous myocardial infarction, previous coronary artery bypass grafting, time between onset of symptoms and first medical contact, randomization to crushed prasugrel, treatment center, culprit vessel on angiography, pre-PCI TIMI flow in the IRA, and pre-PCI thrombus grade in the IRA.

Moreover, an exploratory regression analysis was implemented to assess potential interactions between (1) PCI strategy and thrombus burden in the IRA during initial angiography and (2) between PCI strategy and crushed or integral prasugrel loading dose administration.

## 3. Results

### 3.1. Baseline Characteristics

Table 1 lists the baseline patient characteristics and procedural data. Out of 446 patients, 189 patients (42%) were treated with DS. Patients undergoing DS were younger (60 ± 11 vs. 64 ± 12 years, *p* = 0.001) and less likely to suffer from comorbidities as compared with patients undergoing CS (hypertension: 33% vs. 42%, *p* = 0.047, and previous myocardial infarction: 4% vs. 10%, *p* = 0.020). DS compared with CS was more frequently performed in patients with a thrombotic occlusion of the right coronary artery and less frequently in patients with a thrombotic occlusion of the left anterior descending (50% vs. 36%, *p* = 0.004, and 34% vs. 45%, *p* = 0.021, respectively). Moreover, patients undergoing DS more frequently had pre-PCI TIMI 3 flow in the IRA (before wire crossing) compared with patients undergoing CS (45% vs. 29%, *p* = 0.001) and less frequently had TIMI 0 or 1 flow in the IRA at initial angiography compared with patients undergoing CS (38% vs. 56%, *p* = 0.001). Total procedural time and total fluoroscopy time were both shorter in patients undergoing DS (28 [IQR, 22–39] vs. 36 [IQR, 27–51] minutes, *p* < 0.001, and 6 [IQR, 4–10] vs. 9 [IQR, 6–15] minutes, *p* < 0.001, respectively).

### 3.2. Stent Dimensions

The propensity-score-weighted outcomes of the stent dimensions, early myocardial reperfusion, and clinical outcomes in the complete study population are shown in Table 2. The respective crude outcomes are shown in Supplementary Table S1. In patients undergoing primary PCI, DS patients more often received only one coronary stent in the culprit lesion compared with CS patients (74% vs. 55%, adjusted OR 2.49; [95%CI 1.55–4.01], *p* < 0.001). In line, cumulative stent length in the culprit vessel was shorter in patients who underwent DS compared with CS (23 [IQR, 18–33] vs. 32 [IQR, 23–48] mm, *p* < 0.001, whereas mean stent diameter was similar between groups (3.2 ± 0.5 vs. 3.2 ± 0.5 mm, *p* = 0.17).

### 3.3. Early Myocardial Reperfusion Markers

**Figure 1:**
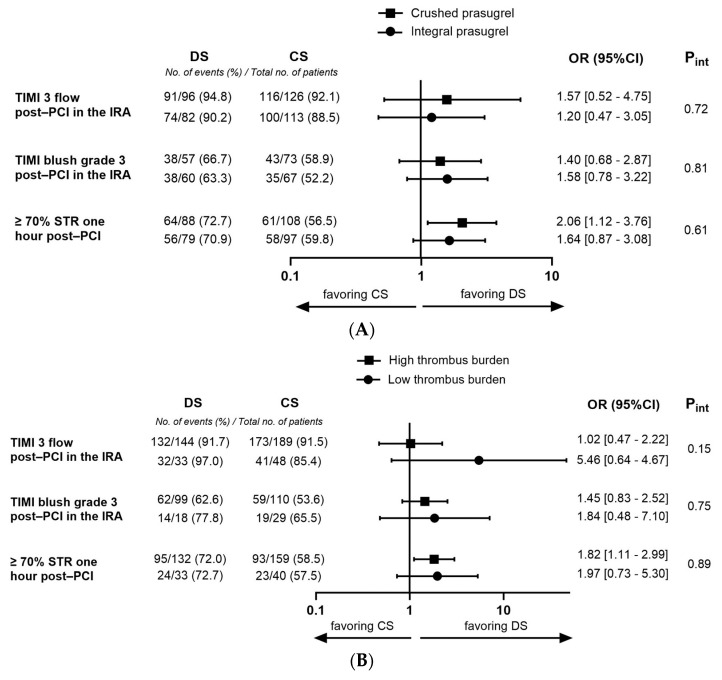
Forest plots visualizing the interaction between PCI strategy and (**A**) crushed or integral prasugrel loading dose administration, and (**B**) high or low thrombus burden on initial angiography. PCI—percutaneous coronary interventions.

TIMI 3 flow in the IRA post-PCI occurred in 165 patients (93%) in the DS group compared with 216 patients (90%) in the CS group (adjusted OR 1.16 [95%CI, 0.56–2.39], *p* = 0.69). Post-PCI TIMI myocardial blush grade 3 was present in 65% of patients undergoing DS versus 56% of patients undergoing CS (adjusted OR 1.31 [95%CI, 0.75–2.31], *p* = 0.35). Median cTFC post-PCI was significantly lower in the DS group compared with the CS group (17 [IQR, 11–25] vs. 19 [IQR, 13–29] FPS, *p* < 0.01). Optimal cTFC was seen in 69% of patients in the DS group versus 62% of patients in the CS group (adjusted OR 1.42 [95%CI, 0.87–2.31], *p* = 0.16). Complete STR one hour post-PCI occurred in 72% of patients in the DS group compared with 58% of patients in the CS group (adjusted OR 1.29 [95%CI, 0.77–2.16], *p* = 0.34). Of note, the effect of DS versus CS on myocardial reperfusion parameters was consistent across the crushed and integral prasugrel loading dose subgroups (Figure 1A).

### 3.4. Clinical Outcomes

At one year, zero patients in the DS group versus eight patients in the CS group died (0% vs. 3%, *p* = 0.02). Three of these deceased patients died from a cardiac cause: one patient died from a free wall rupture on day seven, and two patients died from acute heart failure after 11 and 173 days, respectively. Three patients died from hemorrhagic stroke on days one, seven, and sixteen, respectively. Two patients died from a non-cardiovascular cause of death (sepsis and pulmonary cancer). Stent thrombosis within one year occurred in one patient (0.5%) in the DS group and two patients (0.8%) in the CS group (adjusted OR 0.59 [95%CI, 0.05–6.88], *p* = 0.67). The composite of death or stent thrombosis at one year occurred in one patient (0.5%) in the DS group and ten patients (4.0%) in the CS group (adjusted OR 0.18 [95%CI, 0.02–1.44], *p* = 0.11). Myocardial re-infarction occurred in nine patients (5%) in the DS group versus eight patients (3%) in the CS group (OR 1.86 [95%CI, 0.66–5.26], *p* = 0.24). Out of these cases, four DS patients and three CS patients presented with target lesion failure (2% vs. 1%, adjusted OR 2.93 [95%CI, 0.31–38.93], *p* = 0.31). Other outcomes, including stroke and urgent revascularization rates, did not significantly differ between groups.

### 3.5. Subgroup with High Thrombus Burden at the Beginning of Angiography

A high thrombus burden in the IRA during initial angiography was present in 349 patients (78%). DS was used in 150 of these patients (43%). The baseline and procedural characteristics in patients with high thrombus burden on the initial angiography were comparable to the overall cohort (Supplementary Table S2), with the exception of lower rates of pre-PCI TIMI 3 flow in the IRA in both groups (overall cohort: DS 45% vs. CS 30%, *p* < 0.001, and high thrombus burden cohort: DS 39% vs. CS 21%, *p* < 0.001). The propensity score weighted outcomes of stent dimensions, early myocardial reperfusion, and clinical outcomes in patients presenting with high thrombus burden are shown in Table 3. The respective crude outcomes are described in Supplementary Table S3. The effect of DS on stent usage, early myocardial reperfusion markers, and clinical outcomes in STEMI patients presenting with high thrombus were similar to the observed efficacy of DS in the overall cohort. Furthermore, we found no interactions between PCI strategy and high or low thrombus burdens (Figure 1B).

## 4. Discussion

We conducted a post hoc analysis of the COMPARE CRUSH trial, investigating direct stenting versus conventional stenting in a contemporary cohort of prehospital-managed STEMI patients. Our principal findings were that (1) DS represents a frequently used PCI technique in the contemporary management of STEMI patients in the Netherlands, and (2) there was no significant difference in the occurrence rate of myocardial reinfarction due to target lesion failure during follow-up between DS and CS patients.

The use of DS has significantly increased over the years, indicated by the high use of DS (42%) in the present cohort [14,15]. Increased experience of interventional cardiologists with this technique, combined with adequate prehospital pharmacological therapies preventing clot extension and reformation, might have contributed to the increased safety of DS. The increasing quantity of contradicting data on the efficacy of DS throughout the years has led to a continuing debate about the added value of DS in STEMI patients. The absence of a clear signal toward improved reperfusion and clinical outcomes has caused the European guidelines to refrain from making any recommendations concerning the use of DS [16]. What adds to the complexity of this debate is that only a minority of the available data originates from randomized trials, and most trials were performed in an era before the introduction of several pharmacological and mechanical reperfusion advances (e.g., potent oral P2Y12 inhibitors, second/third generation drug-eluting stents, non-routine use of glycoprotein IIb/IIIa receptor inhibitors, or thrombus aspiration), rendering them no longer generalizable to contemporary practice [17]. Therefore, further research is warranted to determine, in a contemporary treatment setting, whether DS compared with CS is a PCI strategy worth pursuing in that it would lead to more complete early myocardial reperfusion and improved clinical outcomes in STEMI patients undergoing primary PCI.

Balloon-induced barotrauma to the coronary vessel wall during CS has been suggested as a mechanism leading to neo-intimal hyperplasia, thereby increasing the risk of in-stent restenosis [9]. The use of DS—thereby omitting balloon inflations in the infarct-related lesion—was therefore thought to reduce the rate of in-stent restenosis and target lesion failure. However, without preceding balloon inflations, the risk of stent underexpansion and lesion–stent mismatch resulting in stent malposition, as well as the risk of target lesion failure, may increase, according to previous trials [9,18]. Our results show that the cumulative stent length in the culprit vessel was significantly shorter in patients treated with DS compared with CS (23 [18–33] mm vs. 32 [23–48] mm, *p* < 0.001). In this line, we found that DS compared with CS was associated with an independent 2.5-fold ([95%CI, 1.55–4.01], *p* < 0.001) higher chance of successful revascularization with the use of only a single drug-eluting stent. Interestingly, mean stent diameters were comparable irrespective of the stenting strategy used (DS 3.2 ± 0.5 mm vs. CS 3.2 ± 0.5 mm, *p* = 0.17), indicating adequate stent delivery, deployment, and lesion coverage. Furthermore, myocardial reinfarction due to target lesion failure rates did not differ between patients who underwent DS or CS. This lack of a signal toward an increased rate of in-stent restenosis is in line with the results from a coronary imaging sub-study of the CONVERTIBLE trial [19]. That analysis reported quantitative angiographic data at six-month follow-up, which revealed no differences in the mean stent diameter, minimum lumen diameter, or rate of restenosis in patients treated with DS. Our results indicate that, as for stent positioning, diameter, and patency, DS seems comparable to CS.

In our cohort, we saw that after propensity score adjustment, there were no independent early reperfusion differences in terms of ST-segment resolution or TIMI flow between DS and CS patients. Several observational studies have reported signs of improved myocardial tissue reperfusion (expressed as the total infarct size, extent of microvascular obstruction on cardiac magnetic resonance imaging, and complete ST-segment resolution) [5,7,20,21,22]. However, conflicting results came from the randomized DIRAMI trial and a pooled meta-analysis in 2015 [9,17]. The DIRAMI trial included 248 patients presenting with acute myocardial infarction and reported similar incidences of complete STR post-PCI between DS and CS patients. Moreover, Azalini et al. demonstrated that a clear signal toward a benefit of DS compared with CS in terms of improved STR post-PCI was lacking in a pooled meta-analysis including three RCTs and nine observational studies.

Restored epicardial vessel patency at presentation facilitates the use of DS in patients undergoing primary PCI. In this line, several therapies have been investigated as adjunctive therapy for DS, including the use of manual thrombus-aspiration devices and glycoprotein IIb/IIIa inhibitor administration preceding coronary stenting. A large, pooled post-hoc analysis combining three randomized trials investigating the use of routine thrombus-aspiration in STEMI patients failed to substantiate any synergic effect of manual thrombus-aspiration combined with DS [14,23]. In contrast, two small, randomized trials did suggest that thrombectomy preceding coronary stenting can improve the efficacy of DS [24,25]. As for the use of routine, adjuvant glycoprotein inhibitor administration, the available literature has suggested that this adjunctive pharmacologic treatment option is able to provide early epicardial reperfusion at the beginning of primary PCI [26]. Unfortunately, this approach also leads to a significant increase in major bleeding. Moreover, the significant increase in strokes associated with thrombus-aspiration has resulted in the discard of routine use of these strategies for the treatment of STEMI [14]. However, it cannot be ruled out that in certain high-risk patients (e.g., those presenting with a high thrombotic burden at initial presentation), the use of these approaches may add to improved myocardial reperfusion outcomes.

## 5. Limitations

Our results should be interpreted in light of the following limitations. First, this is a post-hoc analysis where treatment was not randomized. Despite our best efforts to minimize the influence of confounders by using a propensity-score-weighted model, our analysis remains susceptible to bias due to unmeasured residual confounding factors. Importantly, several procedural parameters that could have influenced the operator’s choice for stenting strategy (e.g., TIMI flow in the culprit vessel after wire crossing, culprit lesion length and diameter, and the extent of calcification in these lesions) were not available in this sub-study and therefore could not be included in our propensity score. Furthermore, the COMPARE CRUSH trial did not collect data concerning balloon type (compliant versus non-compliant) or data concerning pre- and post-dilatation inflation pressures. Therefore, these results should be interpreted with caution. Second, the sample size of the COMPARE CRUSH cohort was calculated based on its primary myocardial reperfusion endpoints and was therefore not powered to assess clinical outcomes. Third, this trial was conducted in a country with a well-functioning emergency medical service network, where pre-hospital initiation of antithrombotic therapy is the standard of practice. Therefore, our results may not be generalizable to STEMI patients with prolonged transfer times or patients who only receive in-hospital antithrombotic therapy. Moreover, these results cannot be extrapolated to STEMI patients presenting with cardiogenic shock or cardiac arrest.

## 6. Conclusions

In this contemporary STEMI cohort of the randomized COMPARE CRUSH trial, we found that DS is a frequently used interventional technique in the acute phase of STEMI treatment, comprising over 40% of cases in our cohort. We found no difference in the early myocardial reperfusion outcomes between DS and CS. Furthermore, DS seemed comparable to CS in terms of stent diameter and one-year vessel patency.

## Figures and Tables

**Table 1:** Baseline and procedural characteristics in patients treated with direct stenting versus conventional stenting.

	DS (n = 189)	CS (n = 257)	*p*-Value
**Patient characteristics**			
Demographics			
Age—years	60 + 11	64 + 12	0.001
Female sex—no. (%)	49 (25.9)	58 (22.6)	0.41
Caucasian—no. (%)	170 (90.9)/187	234 (92.1)/254	0.65
BMI—kg/m^2^	28 + 5/116	27 + 4/187	0.09
Cardiovascular risk factors—no. (%)			
Hypertension	61 (32.6)/187	106 (41.9)/253	0.047
Diabetes mellitus	28 (15.1)/185	41 (16.1)/254	0.78
Dyslipidemia	37 (20.9)/177	70 (28.6)/245	0.07
Smoking	85 (47.2)/180	107 (43.0)/249	0.38
Family history of cardiovascular disease	70 (38.9)/180	102 (41.8)/244	0.55
Medical history			
Previous PCI	16 (8.5)/189	32 (12.5)/256	0.18
Previous myocardial infarction	8 (4.2)/189	26 (10.2)/256	0.020
Presentation			
Onset symptoms to first medical contact—min	50 [29–122]	65 [34–129]	0.08
Crushed prasugrel—no. (%)	87 (46.0)	120 (46.7)	0.89
**Procedural characteristics**			
Culprit vessel—no. (%)			
LAD	63 (33.9)/186	112 (44.8)/250	0.021
Cx	28 (15.1)/186	43 (17.2)/250	0.55
RCA	93 (50.0)/186	91 (36.4)/250	0.004
Multivessel disease	70 (37.6)/186	117 (46.8)/250	0.07
Procedure			
Onset symptoms to wire crossing—min	132 [103–218]	150 [112–225]	0.11
**Angiographic parameters pre-PCI**			
TIMI 3 flow IRA pre-PCI—no. (%)	82 (44.6)/184	73 (29.2)/250	0.001
TIMI thrombusgrade 3–5—no. (%)	150 (81.1)/185	198 (79.9)/249	0.76
Postdilatation	98 (51.9)	168 (65.4)	0.004
Total procedural time—min	28 [22–39]/187	36 [27–51]/256	<0.001
Total fluoroscopy time—min	6 [4–10]/157	9 [6–15]/229	<0.001

BMI—body mass index; PCI—percutaneous coronary intervention; LAD—left anterior descending; Cx—circumflex; RCA—right coronary artery; TIMI—thrombolysis in myocardial infarction; IRA—infarct-related artery.

**Table 2:** Propensity score weighted reperfusion and clinical outcomes in DS versus CS.

	Odds Ratio (95%CI)	*p*-Value
**Stent dimensions in culprit lesion**		
One DES	2.49 [1.55–4.01]	<0.001
**Early reperfusion parameters post-PCI**		
TIMI 3 flow in the IRA	1.16 [0.56–2.39]	0.69
TIMI blush grade 3	1.31 [0.75–2.31]	0.35
cTFC ≤ 23 frames/s	1,42 [0.87–2.31]	0.16
Complete ST-segment resolution at 1 h	1.29 [0.77–2.16]	0.34
**Clinical outcomes (1 year)**		
All-cause mortality	-	-
Cardiac death	-	-
Myocardial re-infarction	1.86 [0.66–5.26]	0.24
Target lesion failure	2.93 [0.52–16.49]	0.22
Stent thrombosis	0.59 [0.05–6.88]	0.67
Stroke	-	-
Urgent revascularization	2.92 [0.83–10.26]	0.10
Target lesion revascularization	3.49 [0.31–38.93]	0.31
Composite of death and stent thrombosis	0.18 [0.02–1.44]	0.11
Composite of death, myocardial re-infarction, stroke, stent thrombosis, and urgent revascularization	0.93 [0.41–2.10]	0.86

Propensity score included age, gender, Caucasian ethnicity, randomization to crushed prasugrel, weight, height, hypertension, diabetes, dyslipidemia, smoking, family history of cardiovascular disease, previous PCI, previous MI, previous CABG, time between onset of symptoms and FMC, treatment center, culprit vessel on angiography, pre-PCI TIMI flow in the IRA, and pre-PCI thrombusgrade in the IRA. CI—confidence interval; TIMI—thrombolysis in myocardial infarction; IRA—infarct-related artery; PCI—percutaneous coronary intervention.

**Table 3:** Propensity score weighted reperfusion and clinical outcomes of direct stenting versus conventional stenting in patients with high thrombus burden on initial angiography.

	Odds Ratio (95%CI)	*p*-Value
**Stent dimensions in culprit lesion**		
One DES	2.56 [1.49–4.39]	0.001
**Early reperfusion parameters post-PCI**		
TIMI 3 flow in the IRA	0.78 [0.36–1.73]	0.55
TIMI blush grade 3	1.17 [0.64–2.14]	0.60
cTFC ≤ 23 frames/sec	1.51 [0.88–2.59]	0.13
Complete ST-segment resolution	1.34 [0.78–2.30]	0.29
**Clinical outcomes (1 year)**		
All-cause mortality	-	-
Cardiac death	-	-
Myocardial re-infarction	1.92 [0.61–6.03]	0.27
Target lesion failure	3.10 [0.56–17.48]	0.20
Stent thrombosis	1.96 [0.12–31.65]	0.64
Stroke	-	-
Urgent revascularization	3.98 [0.71–22.19]	0.12
Target lesion revascularization	3.68 [0.33–41.16]	0.29
Composite of death and stent thrombosis	0.43 [0.05–3.92]	0.45
Composite of death, myocardial re-infarction, stroke, stent thrombosis, and urgent revascularization	1.23 [0.46–3.32]	0.68

Propensity score included age, gender, Caucasian ethnicity, randomization to crushed prasugrel, weight, height, hypertension, diabetes, dyslipidemia, smoking, family history of cardiovascular disease, previous PCI, previous MI, previous CABG, time between onset of symptoms and FMC, treatment center, culprit vessel on angiography, pre-PCI TIMI flow in the IRA, and pre-PCI thrombus grade in the IRA. CI—confidence interval; TIMI—thrombolysis in myocardial infarction; IRA—infarct-related artery; PCI—percutaneous coronary intervention.

## Data Availability

The data that support the findings of this study are available from the corresponding author on reasonable request.

## Supplementary Materials

The following supporting information can be downloaded at: https://www.mdpi.com/article/10.3390/jcm12206645/s1, Table S1: Crude reperfusion and clinical outcomes in direct stenting versus conventional stenting; Table S2: Baseline characteristics in direct stenting versus conventional stenting patients with high thrombus burden on initial angiography; Table S3: Crude reperfusion and clinical outcomes of direct stenting versus conventional stenting in patients with high thrombus burden on initial angiography.
