# Supplementary material for: Direct Stenting versus Conventional Stenting in Patients with ST-Segment Elevation Myocardial Infarction—A COMPARE CRUSH Sub-Study
Source: J Clin Med. 2023 Oct 20;12(20):6645. doi: 10.3390/jcm12206645 (PMC10607208; doi:10.3390/jcm12206645)
Supplement: Supplementary file 1 [file jcm-12-06645-s001.zip › jcm-2630784-supplementary.pdf]

SUPPLEMENTARY TABLES

**Supplementary table S1**	Crude reperfusion and clinical outcomes in direct stenting versus conventional stenting
**Supplementary table S2**	Baseline characteristics in direct stenting versus conventional stenting patients with high thrombus burden on initial angiography
**Supplementary table S3**	Crude reperfusion and clinical outcomes of direct stenting versus conventional stenting in patients with high thrombus burden on initial angiography

Supplementary table S1. Crude reperfusion and clinical outcomes in direct stenting versus conventional stenting

	DS	CS	Odds ratio (95%CI)	p-value
**Stent dimensions in culprit lesion**				
Cumulative stent length – mm	23 [18 – 33] / 163	32 [23 – 48] / 239	-	<0.001
Diameter – mm	3.22 ± 0.48 / 163	3.15 ± 0.48 / 239	-	0.17
One DES – no. (%)	121 (73.8) / 164	131 (54.6) / 240	2.34 [1.52 – 3.60]	<0.001
**Early reperfusion parameters post-PCI**				
TIMI 3 flow in the IRA – no. (%)	165/178 (92.7)	216/239 (90.4)	1.35 [0.67 – 2.75]	0.41
TIMI blush grade 3 – no. (%)	76/117 (65.0)	78/140 (55.7)	1.47 [0.89 – 2.44]	0.13
cTFC in the IRA – frames/second	17 [11 – 25] / 164	19 [13 – 29] / 215	-	0.02
cTFC ≤ 23 frames/sec – no. (%)	111/160 (69.4)	134/215 (62.3)	1.37 [0.89 – 2.12]	0.16
Complete ST-segment resolution – no. (%)	120/167 (71.9)	119/205 (58.0)	1.85 [1.19 – 2.86]	<0.01
**Clinical outcomes (1 year) – no. (%)**				
All-cause mortality	0/184 (0.0)	8/248 (3.2)	-	0.02*
Cardiac death	0/184 (0.0)	3/248 (1.2)	-	0.27*
Myocardial re-infarction	9/184 (4.9)	8/248 (3.2)	1.54 [0.58 – 4.08]	0.38
Target lesion failure	4/184 (2.2)	3/248 (1.2)	1.81 [0.40 – 8.21]	0.47*
Stent thrombosis	1/184 (0.5)	2/248 (0.8)	0.67 [0.06 – 7.50]	1.00*
Stroke	0/184 (0.0)	3/248 (1.2)	-	0.27*
Urgent revascularization	8/184 (4.3)	5/248 (2.0)	2.21 [0.71 – 6.87]	0.17
Target lesion revascularization	2/184 (1.1)	2/248 (0.8)	1.35 [0.19 – 9.69]	1.00*
Composite of death and stent thrombosis	1/184 (0.5)	10/248 (4.0)	0.13 [0.02 – 1.03]	0.054
Composite of death, myocardial re-infarction, stroke, stent thrombosis and urgent revascularization	11/184 (6.0)	18/248 (7.3)	0.80 [0.37 – 1.76]	0.60

* p-value calculated with Fisher's exact test

CI – confidence interval; TIMI – thrombolysis in myocardial infarction; IRA – infarct-related artery; PCI – percutaneous coronary intervention; cTFC – corrected TIMI frame count.

Supplementary table S2. Baseline characteristics of direct stenting versus conventional stenting in patients with high thrombus burden on initial angiography

	DS (n=150)	CS (n=199)	p-value
**Patient characteristics**			
Demographics			
Age – years	60 + 12	64 + 11	0.007
Female sex – no. (%)	39 (26.0)	45 (22.6)	0.46
Caucasian – no. (%)	133 (89.3) / 149	182 (92.4) / 197	0.31
BMI - kg/m^2	28 + 5 / 95	27 + 4 / 140	0.29

Cardiovascular risk factors – no. (%)			
Hypertension	47 (31.5) / 149	77 (39.5) / 195	0.13
Diabetes mellitus	25 (17.0) / 247	32 (16.2) / 197	0.85
Dyslipidemia	30 (21.4) / 140	49 (25.8) / 190	0.36
Smoking	65 (45.5) / 143	83 (43.0) / 193	0.66
Family history of cardiovascular disease	51 (35.9) / 142	82 (43.6) / 188	0.16
Medical history			
Previous PCI	12 (8.0) / 150	24 (12.1) / 198	0.21
Previous myocardial infarction	7 (4.7) / 150	19 (9.6) / 198	0.08
Presentation			
Onset symptoms to first medical contact – min	50 [29 – 109]	64 [30 – 116]	0.09
Crushed prasugrel – no. (%)	68 (45.3)	95 (47.7)	0.66
**Procedural characteristics**			
Culprit vessel – no. (%)			
LAD	49 (32.7) / 150	81 (40.9) / 198	0.12
Cx	23 (15.3) / 150	37 (18.7) / 198	0.41
RCA	76 (50.7) / 150	78 (39.4) / 198	0.036
Multivessel disease – no. (%)	62 (41.3) / 150	91 (45.7) / 198	0.41
**Angiographic parameters pre-PCI**			
Onset symptoms to wire crossing – min	131 [101 – 192]	150 [111 – 220]	0.06
TIMI 3 flow IRA pre-PCI – no.(%)	58 (39.2) / 148	41 (20.6) / 199	<0.001
Postdilatation	75 (50.0)	124 (62.3)	0.021
Total procedural time – min	28 [23 – 39]	35 [27 – 49]	<0.001

BMI – body mass index; PCI – percutaneous coronary intervention; LAD – left anterior descending; Cx – circumflex; RCA – right coronary artery; TIMI – thrombolysis in myocardial infarction; IRA – infarct-related artery.

Supplementary table S3. Crude reperfusion and clinical outcomes of direct stenting versus conventional stenting in patients with high thrombus burden on initial angiography

	DS	CS	Odds ratio (95%CI)	p-value
**Stent dimensions in culprit lesion**				
Cumulative stent length – mm	23 [28 – 31]	32 [23 – 47]	-	<0.001
Diameter - mm	3.23 + 0.48	3.15 + 0.50	-	0.18
One DES – no. (%)	100/129 (77.5)	107/184 (58.2)	2.48 [1.50 – 4.12]	<0.001
**Early reperfusion parameters post-PCI**				
TIMI 3 flow in the IRA – no. (%)	132 (91.7)	173 (91.5)	1.02 [0.47 – 2.22]	0.97
TIMI blush grade 3 – no. (%)	62 (62.6)	59 (53.6)	1.45 [0.83 – 2.52]	0.19
cTFC – frames/second	16 [12 – 24]	20 [13 – 29]	-	0.018
cTFC ≤ 23 frames/sec – no. (%)	92/129 (71.3)	106/175 (60.6)	1.62 [0.99 – 2.64]	0.053
Complete ST-segment resolution – no. (%)	95 (72.0)	93 (58.5)	1.82 [1.11 – 2.99]	0.017
**Clinical outcomes (1 year)** – no. (%)				
All-cause mortality	0/147 (0.0)	3/193 (1.6)	-	0.26*

Cardiac death	0/147 (0.0)	1/193 (0.5)	-	1.00*
Myocardial re-infarction	7/147 (4.8)	7/193 (3.6)	1.33 [0.46 – 3.87]	0.60
Target lesion failure	4/147 (2.7)	3/193 (1.6)	1.73 [0.38 – 7.87]	0.47*
Stent thrombosis	1/147 (0.7)	2/193 (1.0)	0.65 [0.06 – 7.28]	1.00*
Stroke	0/147 (0.0)	1/193 (0.5)	-	1.00*
Urgent revascularization	5/147 (3.4)	3/193 (1.6)	2.23 [0.52 – 9.49]	0.30*
Target lesion revascularization	2/147 (1.4)	2/193 (1.0)	1.32 [0.18 – 9.46]	1.00*
Composite of death and stent thrombosis	1/147 (0.7)	5/193 (2.6)	0.26 [0.03 – 2.22]	0.24*
Composite of death, myocardial re-infarction, stroke, stent thrombosis and urgent revascularization	8/147 (5.4)	11/193 (5.7)	0.95 [0.37 – 2.43]	0.92

* p-value calculated with Fisher's exact test

DS – direct stenting; CS – conventional stenting; CI – confidence interval; TIMI – thrombolysis in myocardial infarction; IRA – infarct-related artery; PCI – percutaneous coronary intervention; cTFC – corrected TIMI frame count.
